# Cyclin A is a prognostic indicator in early stage breast cancer with and without tamoxifen treatment

**DOI:** 10.1038/sj.bjc.6600072

**Published:** 2002-02-01

**Authors:** R Michalides, H van Tinteren, A Balkenende, J B Vermorken, J Benraadt, J Huldij, P van Diest

**Affiliations:** Division of Tumour Biology, the Netherlands Cancer Institute, Plesmanlaan 121, 1066CX Amsterdam, The Netherlands; Department of Biometrics, the Netherlands Cancer Institute, Plesmanlaan 121, 1066CX Amsterdam, The Netherlands; Department of Medical Oncology, University Hospital of Antwerp, Wilrijkstraat 10, 2650 Edegem, Belgium; Comprehensive Cancer Center Amsterdam, Plesmanlaan 125, 1066CX Amsterdam, The Netherlands; Department of Pathology, VU Medical Center, PO Box 7057, NL-1007 MB Amsterdam, The Netherlands

**Keywords:** cyclin A, cyclin D1, human breast cancer, prognosis, tamoxifen, cell cycle markers

## Abstract

Overexpression of G1-S regulators cyclin D1 or cyclin A is frequently observed in breast cancer and is also to result in ligand-independent activation of oestrogen receptor *in vitro*. This might therefore, provide a mechanism for failure of tamoxifen treatment. We examined by immunohistochemical staining the effect of deregulation of these, and other cell cycle regulators on tamoxifen treatment in a group of 394 patients with early stage breast cancer. In univariate analysis, expression of cyclin A, Neu, Ki-67 index, and lack of OR expression were significantly associated with worse prognosis. When adjusted by the clinical model (for lymph node status, age, performance status, T-classification, grade, prior surgery, oestrogen receptor status and tamoxifen use), only overexpression of cyclin A and Neu were significantly associated with worse prognosis with hazard ratios of, respectively, 1.709 (*P*=0.0195) and 1.884 (*P*=0.0151). Overexpression of cyclin A was found in 86 out of the 201 OR-positive cases treated with tamoxifen, and was the only independent marker associated with worse prognosis (hazard ratio 2.024, *P*=0.0462). In conclusion, cyclin A is an independent predictor of recurrence of early stage breast cancer and is as such a marker for response in patients treated with tamoxifen.

*British Journal of Cancer* (2002) **86**, 402–408. DOI: 10.1038/sj/bjc/6600072 www.bjcancer.com

© 2002 The Cancer Research Campaign

## 

The signalling pathway of oestrogen receptor-α, OR-α, is a major determinant in human breast tumorigenesis (Brown *et al*, 1999). This assumption is derived from observations that OR-α is only present in a minority of normal breast epithelial cells, whereas >70% of human breast cancer contain OR-α (Ricketts, 1991). Moreover, oestrogen causes proliferation of OR positive breast cancer cells (Chalbos *et al*, 1982). The mitogenic activity of oestrogen is still far from being fully understood. Oestrogen induces rapid changes in cell cycle progression kinetics and results in altered expression of cell cycle markers in oestrogen responsive cells (Musgrove *et al*, 1993; Altucci *et al*, 1996; Foster and Wimalasena, 1996). These involve key transitions in the eukaryotic cellular division, which are controlled by the sequential activation and inactivation of cyclin dependent protein kinases (cdk). The activity of these kinases is regulated by phosphorylation/dephosphorylation, subcellular traffic mechanism, association with cyclin proteins and with specific cdk-inhibitors, and by proteolytic degradation (Sherr and Roberts, 1999). Of these cyclin-cdk complexes, cyclin D-cdk4/6 activity drives cells through the early G1 phase of the cell cycle, whereas cyclin E-cdk2 and subsequently cyclin A-cdk2 activities are required for transition through the later G1 phase of the cell cycle past the restriction point up to which growth factor stimulation is mandatory. The mitogenic activity of oestrogen involves stimulation of expression of cyclin D1 (Altucci *et al*, 1996; Sabbah *et al*, 1999) and of cdc25A (Foster *et al*, 2001) an activating phosphatase of cdk2 (Jinno *et al*, 1994). Inhibition of oestrogen receptor activity by anti-oestrogens leads to reduced cyclin D1 expression and, as a consequence thereof, to a release of cdk-inhibitors p21 and p27 from the cyclin D-cdk4/6 complex that then become associated with cyclin-cdk2 complexes. This shift of cdk-inhibitors results in a cell cycle block in mid-G1 (Planas-Silva and Weinberg, 1997; Prall *et al*, 1997). A direct effect of anti-oestrogens on effectors of the cell cycle downstream of cyclin D1 can, however, not be excluded (Cicatiello *et al*, 2000; Foster *et al*, 2001).

Anti-oestrogens compete with oestrogen for binding to the OR, but fail to induce receptor activation. These molecules interfere with multiple steps in the OR signalling pathway, including dissociation from chaperone proteins, nuclear translocation, dimerization and targeting to OR specific DNA elements and binding to transcriptional co-regulators (Osborne *et al*, 2000). Because of their ability to disrupt OR signalling pathways, anti-oestrogens are widely used for the treatment of hormone-dependent breast cancer. The non-steroidal triphenyl-ethylene anti-oestrogen tamoxifen has been established as the first choice for adjuvant therapy of oestrogen-receptor positive breast cancer (Early Breast Cancer Trialist's Collaborative Group, 1998; Yao and Jordan, 1999).

OR-α signalling activity is not only induced by oestrogen, but OR-α is also activated as a transcriptional transactivator in a hormone-independent manner by phosphorylation of serines 104/106 by cyclin A-cdk2 (Rogatsky *et al*, 1999) and by direct binding of OR-α to cyclin D1 (Zwijsen *et al*, 1997). Both of these modulations affect the association between OR-α and co-activators of OR-α (Rogatsky *et al*, 1999; Zwijsen *et al*, 1999) and may thereby affect the ability of anti-oestrogens to inhibit OR-α activity. It has, therefore, been hypothesized that tamoxifen treatment of breast cancer may be inefficient in tumour cases that harbour genetic lesions which result in elevated levels of cyclin D1 or A, or that lead to reduced levels of cdk inhibitors p21 or p27 (Michalides, 1999; Cariou *et al*, 2000). We have addressed this hypothesis by examining the effect of an altered expression of these cell cycle regulators on the outcome of tamoxifen treatment of patients with early stage of breast cancer.

## MATERIALS AND METHODS

### Patients and tumour specimens

**Figure 1:**
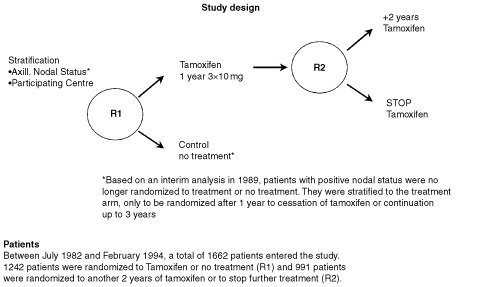
Design of the IKA Tamoxifen study.

The marker group of 394 patients is part of a clinical Comprehensive Cancer Center Amsterdam (IKA) Tamoxifen study on the effect of adjuvant tamoxifen treatment in breast cancer. Between 1982 and 1994, 1662 patients were randomized in a 2 : 1 distribution to control or tamoxifen (30 mg day^−1^). Patients were eligible if they were post-menopausal, less than 76 years of age and had a T_1–4_, N_0–3,_ M_0_ breast tumour (World Health Organization, 1981), but no mastitis or palpable supra- or infraclavicular lymph nodes. Randomization was stratified by institute and nodal status (N+/N−). After 1989, based on two interim analyses showing a significant improvement in progression-free survival in node positive patients, these patients skipped the first randomization and all received 1 year of tamoxifen. After 1 year they were again eligible for the second randomization in the study to receive either another 2 years of tamoxifen or to stop further treatment (Figure 1

). The patient characteristics and clinical outcome of tamoxifen treatment of the original study group (1662 patients) have been presented elsewhere (Vermorken *et al*, 1998). Of the patients in this trial, paraffin embedded material was available from a randomly sampled group of 394 patients. Tissues had been fixed for at least 24 h in neutral buffered 4% formaldehyde. After paraffin embedding, 4-μM-thick sections were cut and attached on silane-coated slides. All tumours used in the marker study were examined by one pathologist (PvD), were classified according to WHO criteria (World Health Organization, 1981) and were graded according to a modification of Bloom and Richardson's method (Elston and Ellis, 1990). During the clinical study, oestrogen receptor status was determined routinely by the dextran-coated charcoal assay on tumour cytosols. Receptor levels of >10 fmol mg^−1^ of cytosolic protein were considered positive. Tumours were also examined for oestrogen receptor by immunohistochemistry.

### Immunohistochemistry

Immunohistochemistry was performed as described previously (Michalides *et al*, 1996; Van Diest *et al*, 1997). In all cases, except for staining with anti-Neu, antigen retrieval was applied by microwave treatment in citrate buffer pH 6. Slides were blocked with 10% normal serum, depending on the first antiserum to be used, and were incubated overnight with the following antisera and dilutions: from Novacastra (Newcastle, UK): cyclin D1 (clone DCS-6, 1 : 40); cyclin A (clone 6E6, 1 : 200), epidermal growth factor receptor, EGF-R (clone EGFR.113, 1 : 20); from Pharmingen (San Diego, USA): p21 (clone 6B6, 1 : 500); from Transduction Laboratories (Lexington, USA): p27 (clone K2052, 1 : 200); from Dako (Glostrup, Denmark): OR (clone OR1D7, 1 : 50), progesterone receptor, PgR (cl PgR 636, 1 : 100), p53 (clone DO-7, 1 : 500); from Immunotech SA (Marseille, France): Ki-67 (Mib1, 1 : 40). Anti-Neu antibody was obtained from M vd Vijver (The Netherlands Cancer Institute, Amsterdam, The Netherlands) and was used at a dilution of 1 : 1000. All first antibodies were incubated overnight at 4°C, except for anti-EGFR that was incubated at room temperature.

Biotinylated second antibodies (Dako, Glostrup, Denmark) were applied for 30 min at room temperature, after which peroxidase-conjugated streptavidin-biotin labelling and subsequent DMBA staining was performed.

The slides were independently examined by two observers (PvD and RM). For staining of EGFR and Neu only, membrane staining was considered as positive staining. With the other markers, cells were considered to be positive only when distinct nuclear staining was identified. The percentage of immunoreactive cells was evaluated by scanning whole sections at medium and high magnifications. p27 expression was scored as low (<50% reacting cells) and high (>50%). Scores for positivity for cyclins D1 and E, OR, PgR, Ki-67, p21 and p53 were determined semi-quantitatively as described in Materials and Methods. The threshold for positivity for cyclin A staining was determined from analyzing the Martingale residuals of cyclin A positives with recurrence as the outcome. Above 5%, which was also the median value of cyclin A, excess in risk for recurrence was observed. The range of cyclin A positive cells in this series ranged from 0–50%, with an average of 9.5%. Cyclin D1, OR, PgR and Ki-67 were considered positive if >5% of the cells stained, for cyclin A expression >10% of the cells staining was considered as positive. All cases expressing cyclin E, p21 and p53 were considered positive, this in accordance with earlier settings of (Michalides *et al*, 1996; Van Diest *et al*, 1997; Barbareschi *et al*, 2000).

### Statistical analysis

The endpoint for analysis was recurrence, defined as the first reappearance of breast cancer at any site (local, contralateral, or distant). Survival estimates and curves were calculated with the Kaplan-Meier technique and differences in time to recurrence were tested by means of a log-rank test. To estimate the association of markers with time to recurrence adjusted for other (known) clinically important variables, Cox proportional hazard analysis was used. The model for adjustment consisted of the following variables: lymph node status (N0, N+), Age (continuous), Karnofski performance index (<80, 80–100), T-classification (T1, T2, T3, T unknown), histological grade (grade I, grade II, grade III), OR status (OR+, OR-, OR unknown), and breast conserving surgery or mastectomy. The association between each of the clinicopathological markers individually was analyzed by Spearman rank correlation.

## RESULTS

Baseline characteristics of the IKA tamoxifen trial patients who also participated in the marker study are shown in Table 1

**Table 1:**
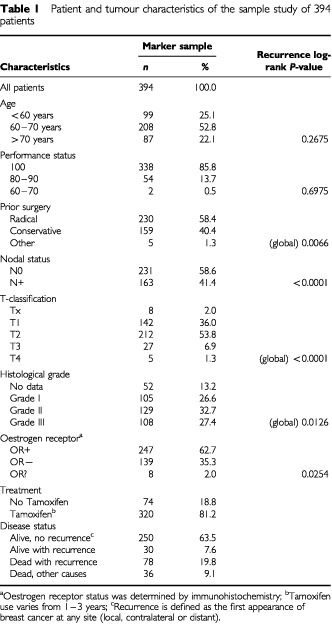
Patient and tumour characteristics of the sample study of 394 patients

. The median follow-up is almost 10 years in the tamoxifen study and over 8 years in the marker study. In 108 patients of the 394 patients in the marker study (27%), breast cancer reappeared. The associations between clinical and pathological parameters and recurrence were as to be expected. The great majority of the 394 patients (320; 81%) had received tamoxifen. Tamoxifen showed to be significantly associated with a better prognosis in the original trial group of 1662 patients (hazard ratio (HR)=0.66, 95% CI 0.55–0.80, *P*=0.0001), but a similar effect seen in the marker study group of 394 patients was not significant (HR=0.66, 95% CI 0.39–1.13, *P*=0.127).

The results of the marker studies and their association with time to recurrence are presented in Tables 2

**Table 2:**
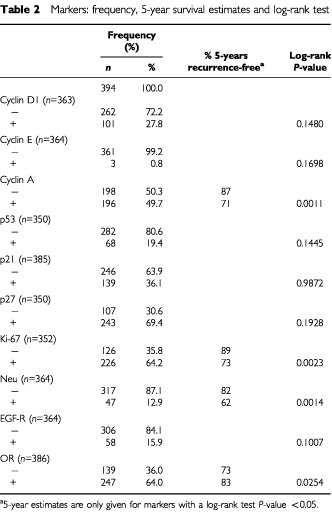
Markers: frequency, 5-year survival estimates and log-rank test

and 3

**Table 3:**
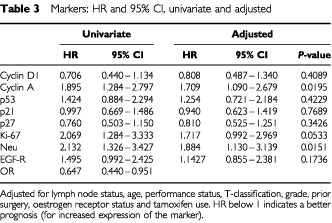
Markers: HR and 95% CI, univariate and adjusted

**Figure 2:**
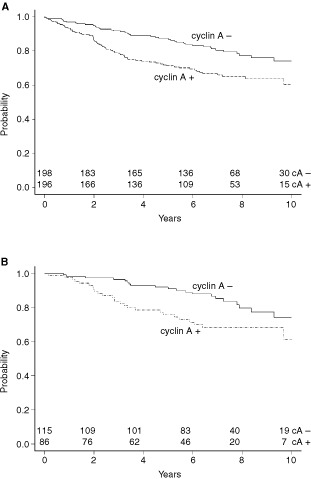
Recurrence-free interval curves for groups of breast cancer patients (**A**) with and without overexpression of cyclin A, and (**B**) OR-positive breast cancer treated with tamoxifen, with and without overexpression of cyclin A (log rank *P* value=0.0462).

. Nuclear staining was also observed together with a weak positive cytoplasmic staining in some of the cyclin A positive tumour cells. This may well indicate cells at G2 phase of the cell cycle. In univariate analysis, expression of cyclin A and Neu, high Ki-67 index, and lack of OR expression were significantly associated with worse prognosis. When adjusted by the clinical model (for lymph node status, age, performance status, T-classification, grade, prior surgery, oestrogen receptor status and tamoxifen use), only overexpression of cyclin A and Neu were significantly associated with worse prognosis with HR of, respectively, 1.709 (*P*=0.0195) and 1.884 (*P*=0.0151), see also Figure 2

. None of the other markers used were independent indicators of prognosis, including cyclin D1, cyclin E, p53, p21, p27, and EGF-R.

Table 4

**Table 4:**
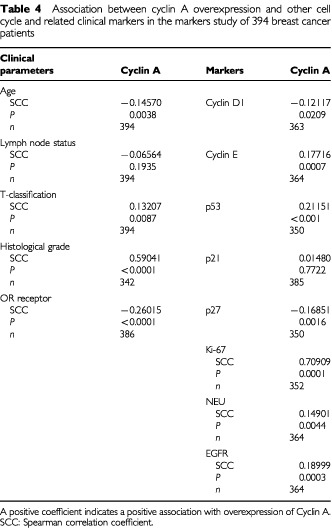
Association between cyclin A overexpression and other cell cycle and related clinical markers in the markers study of 394 breast cancer patients

summarizes the relationship between cyclin A expression and the other markers.

**Figure 3:**
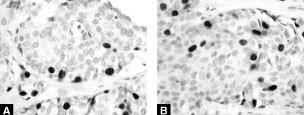
(**A**, **B**) Immunohistochemical staining of breast cancer with antibody 6E6 specific for cyclin A. Note the nuclear staining with a weak cytoplasmic staining in some of the cyclin A positive tumour cells that may indicate cells at G2 phase of the cell cycle.

Cyclin A overexpression was significantly associated with overexpression of Neu, Ki-67, with expression of p53, with absence of OR, with high histological grade and high T-classification. Altogether, these associations suggest that overexpression of cyclin A is more frequently found in large size, undifferentiated, OR-negative breast tumours with an increased proliferative fraction. A representative cyclin A staining in breast cancer cells is given in Figure 3

.

To analyze which of the markers studied contributed to a more inefficient treatment with tamoxifen, we examined a more restricted group of OR positive breast cancer patients treated with tamoxifen, presuming that any effect should be eminent here. Overexpression of cyclin A was the only independent marker associated with a worse prognosis in tamoxifen treated OR-positive tumours, with a HR of 2.024 (*P*=0.0462). Cyclin A overexpression was found in 86 out of 201 OR-positive cases that were treated with tamoxifen.

## DISCUSSION

Cyclin D1 and cdc25A behave as oestrogen-sensitive oncogenes (Altucci *et al*, 1996; Sabbah *et al*, 1999; Foster *et al*, 2001). Introduction of these genes in murine fibroblasts contributes to cellular transformation, and genetic alterations leading to increased expression of these genes are frequently observed in breast cancer (Quelle *et al*, 1993; Galaktionov *et al*, 1995; Michalides, 1999; Cangi *et al*, 2000). Mice transgenic for cyclin D1 are prone to tumour development, the type of tumour being dependent on the enhancer sequence used to construct the cyclin D1 transgene (Bodrug *et al*, 1994; Wang *et al*, 1994; Robles *et al*, 1996). However, neither overexpression of cyclin D1 or of cdc25A was found to be an independent indicator of prognosis in early stage breast cancer (Michalides *et al*, 1996; Van Diest *et al*, 1997; Cangi *et al*, 2000; Leong *et al*, 2000). The present study also indicates that overexpression of cyclin D1 does not affect the outcome of tamoxifen treatment of early stage, OR-α positive breast cancer. Although we did not study directly expression of cdc25A in these tumours, we found that overexpression of cyclin A is significantly associated with worse outcome in tamoxifen treated patients (*P*=0.0462). In the whole group of patients, including 247 OR-positive, 139 OR-negative and eight OR-unknown cases, overexpression of cyclin A and of Neu were indicative of worse outcome, with *P* values of 0.0195 and 0.0151 respectively (Table 3). Neu overexpression already has previously been reported to be associated with a worse prognosis (Slamon *et al*, 1987) and more recently was found to be indicative of tamoxifen resistance (Houston *et al*, 1999; Stal *et al*, 2000). The prognostic value of cyclin A overexpression may well be contributed to its interaction with large sized, undifferentiated, OR-negative breast tumours with an increased proliferative capacity (Table 4). However, also in the group of OR-α-positive breast cancers, cyclin A overexpression did behave as an independent marker of worse prognosis. In our study, overexpression of p53, indicative of p53 mutation, was not associated with outcome of disease or tamoxifen treatment. Only specific p53 mutations, determined by sequencing of the p53 gene, are most informative in predicting response to systemic therapy of advanced breast cancer (Berns *et al*, 2000), whereas we examined early stage breast cancer by p53 immunohistochemistry, which is less accurate in detecting mutations.

We hypothesized that cyclin D1 and/or cyclin A might affect outcome of tamoxifen treatment, since both cyclins are hormone-independent activators of OR-α in *in vitro* experiments (Zwijsen *et al*, 1997; Rogatsky *et al*, 1999). Cyclin D1 does so by binding to OR-α and thereby enhances the interaction between OR-α and SRC (steroid receptor coactivator)-1 (Zwijsen *et al*, 1999), whereas cyclin A-cdk2 phosphorylates cdk2 at serines 104/106 (Rogatsky *et al*, 1999), and thereby activates OR-α transcriptional activity. The activation of OR-α by these cyclins is tamoxifen-insensitive, but this depended on *in vitro* transient transfection where excess cyclin proteins are being generated. However, an experimentally induced six-fold overexpression of cyclin D1 in MCF-7 cells did not render these cells to grow in the presence of anti-oestrogens (Pacilio *et al*, 1998). That finding and the present study indicate that overexpression of cyclin D1 as it is being observed under experimental *in vitro* transfection conditions, is not likely to be encountered *in vivo*. This may only be achieved during lactation where co-stimulation of protein kinase A together with oestrogens does result in excessive levels of cyclin D1 that activate OR (Lamb *et al*, 2000). It should also be mentioned that cyclin D1 is stimulating OR transcriptional activity only when it is free of its regular cdk4/6 partner (Zwijsen *et al*, 1997), but that free cyclin D1 is more prone to proteolytic degradation than when bound to cdk4/6 (Diehl *et al*, 1997). Another confounding factor may be the activation by cyclin D1 of p21 (Barbareschi *et al*, 1997; De Jong *et al*, 1999).

We applied the immunohistological stainings of cell cycle related markers on the residual samples available from the original IKA Tamoxifen study of 1662 patients. In the cohort of 394 patients which was available for the marker study, tumour recurrence was still associated with the base-line clinical prognostic parameters such as grade, stage and lymph node involvement, as was the case in the original study including 1662 patients, indicating that the sample used for the present study was representative (Vermorken *et al*, 1998). A drawback of this study was the relatively short period of tamoxifen treatment of the patients during 1 or 3 years, according to the original setting up of the trial. Recent studies suggest that tamoxifen should be given for longer periods (probably 5 years or more) (Early Breast Cancer Trialist's Collaborative Group, 1998; Fischer *et al*, 2001; Steward *et al*, 2001). In our marker study about 50% of the patients received tamoxifen for only 1 year and 74 patients did not receive tamoxifen treatment at all. This shorter period of tamoxifen treatment has most likely influenced our results in narrowing the effective window of tamoxifen treatment. Nevertheless, some putative prognostic indicators for treatment could be identified in this group of tamoxifen-treated patients.

Overexpression of cyclin D1 is generally found in OR-α-positive, more differentiated breast cancer (Gillett *et al*, 1996; Michalides *et al*, 1996; Van Diest *et al*, 1997), whereas overexpression of cyclin A is associated with OR-α-negative, undifferentiated breast cancer (this study). In both cases overexpression of the cyclins is associated with higher proliferation index (Ki-67 staining). The effect of overexpression of cyclin D1 on the outcome of tamoxifen treatment is controversial: overexpression of cyclin D1 messenger RNA was found to be predictive of poor prognosis in lymph node positive, OR-α-positive breast cancer (Kenny *et al*, 1999), which would support the hypothesis raised above. However, other reports indicate that moderate/strong staining of cyclin D1 protein was associated with complete or partial response to tamoxifen treatment (Fischer *et al*, 2001). Our study shows no effect of cyclin D1, neither on the incidence of recurrence, as was also reported previously (Michalides *et al*, 1996), nor on outcome of tamoxifen treatment within the OR-α-positive group of patients. Since cyclin D1 staining is associated with OR positivity, overexpression of cyclin D1 is likely indicating a beneficial response to tamoxifen when all breast cancers, OR-positive and -negative, are taken together.

Overexpression of cyclin A is associated with undifferentiated, OR-negative and Ki-67 positive breast tumours, which are general features of a more aggressive breast tumour phenotype. Overexpression of cyclin A protein may represent an increased growth fraction of the tumour, an elevated expression of cyclin A protein per tumour cell, or a combination of both. The end result, increased positivity of a tumour for cyclin A staining, is associated with poor clinical course. Since cyclin A associated kinase activity is stimulated by cdc25A, the effect of overexpression of cyclin may well be redundant with that of cdc25A (Blomberg and Hoffmann, 1999). Interestingly, overexpression of cdc25A is indicative of a weakly increased risk of dying of disease (Cangi *et al*, 2000), whereas cyclin A is a significant prognostic indicator of poor prognosis, for OR-positive as well as for OR-negative breast cancer (Bukholm *et al*, 2001; this study).

These results and those of others (Nielsen *et al*, 1999) indicate that defects in G1 regulation are involved in breast cancer progression, and that particular defects are indicative of prognosis and of response to tamoxifen therapy. The relatively weak statistical significance of these associations with prognosis suggests, however, that other interactive factors are involved. This study and that of Cangi *et al* (2000) provide evidence that the anti-mitogenic effects of anti-oestrogens are also *in vivo* to be overcome by mechanisms that affect cdk2 activation and cyclin A expression. Patients with such a primary breast cancer may therefore, benefit from other treatments, for instance from chemotherapy or potential cdk2 inhibitors. We found that cyclin A overexpression is indicative of a poor prognosis in early stage breast cancer and may therefore, serve to further differentiate therapy in both tamoxifen-treated and non-treated patients.

## Appendix

Participating hospitals in the IKA trial (CKTO 8209) were: Medisch Centrum Alkmaar, Alg. Chr. Ziekenhuis Eemland (Amersfoort), Academisch Medisch Centrum (Amsterdam), Antoni van Leeuwenhoek Ziekenhuis (Amsterdam), Onze Lieve Vrouwe Gasthuis (Amsterdam), Vrije Universiteit Medisch Centrum (Amsterdam), Rode Kruis Ziekenhuis (Beverwijk), Ziekenhuis Gooi Noord (Blaricum), Gemini Ziekenhuis (Den Helder), Deventer Ziekenhuis, Albert Schweitzer Ziekenhuis (Dordrecht), Kennemer Gasthuis (Haarlem), Streekziekenhuis Coevorden-Hardenberg (Hardenberg), Spaarne Ziekenhuis (Heemstede), Streekziekenhuis Midden-Twente (Hengelo), Ziekenhuis Hilversum, Westfries Gasthuis (Hoorn), IJsselmeerziekenhuizen (Lelystad), St. Antonius Ziekenhuis (Nieuwegein), Medisch Spectrum Twente, locatie Oldenzaal, Waterlandziekenhuis (Purmerend), Diakonessenhuis (Utrecht), Universitair Medisch Centrum Utrecht, Streekziekenhuis Koningin Beatrix (Winterswijk).
